# Disposal Immunosensor for Sensitive Electrochemical Detection of Prostate-Specific Antigen Based on Amino-Rich Nanochannels Array-Modified Patterned Indium Tin Oxide Electrode

**DOI:** 10.3390/nano12213810

**Published:** 2022-10-28

**Authors:** Liang Yan, Shuai Xu, Fengna Xi

**Affiliations:** 1 Shanxi Bethune Hospital, Shanxi Academy of Medical Sciences, Tongji Shanxi Hospital, Third Hospital of Shanxi Medical University, Taiyuan 030032, China; 2 Tongji Hospital, Tongji Medical College, Huazhong University of Science and Technology, Wuhan 430030, China; 3 Department of Chemistry, Zhejiang Sci-Tech University, Hangzhou 310018, China

**Keywords:** disposable immunosensor, electrochemical detection, nanochannel array, patterned ITO electrode, prostate-specific antigen

## Abstract

Sensitive detection of prostate-specific antigens (PSA) in serum is essential for the prevention and early treatment of prostate cancer. Simple and disposable electrochemical immunosensors are highly desirable for screening and mobile detection of PSAs in high-risk populations. Here, an electrochemical immunosensor was constructed based on amino-rich nanochannels array-modified patterned, inexpensive, and disposable indium tin oxide (ITO) electrodes, which can be employed for the sensitive detection of PSA. Using an amino-group-containing precursor, a vertically ordered mesoporous silica nanochannel film (VMSF) containing amino groups (NH_2_-VMSF) was rapidly grown on ITO. When NH_2_-VMSF contained template surfactant micelle (SM), the outer surface of NH_2_-VMSF was directionally modified by aldehyde groups, which enabled further covalent immobilization of the recognitive antibody to prepare the immuno-recognitive interface. Owing to the charge-based selective permeability, NH_2_-VMSF can electrostatically adsorb negatively charged redox probes in solution (Fe(CN)_6_^3−/4−^). The electrochemical detection of PSA is realized based on the mechanism that the antigen–antibody complex can reduce the diffusion of redox probes in solution to the underlying electrode, leading to the decrease in electrochemical signal. The constructed immunosensor can achieve sensitive detection of PSA in the range from 10 pg/mL to 1 μg/mL with a limit of detection (LOD) of 8.1 pg/mL. Sensitive detection of PSA in human serum was also achieved. The proposed disposable immunosensor based on cheap electrode and nanochannel array is expected to provide a new idea for developing a universal immunosensing platform for sensitive detection of tumor markers.

## 1. Introduction

As one of the most common malignant tumors in men worldwide, prostate cancer, an epithelial malignancy of the prostate gland, is regarded as the “invisible killer” of middle-aged and elderly men [1,2]. This is because patients with early-stage prostate cancer can achieve good therapeutic effects or be cured by radical surgery or radiotherapy. However, the early symptoms of prostate cancer are not obvious, and the fatality rate is high when found in the late stage. Therefore, early prevention, early diagnosis, and early treatment are the keys to improving the survival rate of prostate cancer. The clinical diagnosis of prostate cancer mainly relies on digital rectal examination, detection of prostate-specific antigen (PSA) in serum, prostate ultrasound (transrectal), and pelvic magnetic resonance imaging (MRI). Amongst these, the detection of PSA in serum has the advantages of non-invasiveness and easy operation, demonstrating great potential in screening of high-risk groups, early diagnosis, and monitoring of curative effect [3,4]. As a tumor biomarker secreted by prostate epithelial cells, PSA exists in prostate tissue and semen, and is present in very low levels in normal human serum. PSA in normal human serum is generally less than 4 ng/mL. A higher PSA level of 4-10 ng/mL is called the gray zone for prostate cancer diagnosis. Prostate cancer patients commonly have a PSA level higher than 10 ng/mL [5]. As PSA has extremely high tissue specificity, it has become the preferred biomarker for the diagnosis of prostate cancer. The development of a simple and low-cost method for sensitive detection of PSA in serum is of great significance for non-invasive screening and early diagnosis of prostate cancer.

Until now, immunoassays, including enzyme-linked immunoassay (ELISA), chemiluminescence immunoassay, and electrochemiluminescence (ECL) immunoassay based on magnetic bead, were mainly applied for the detection of PSA [6,7,8,9]. However, these strategies mostly employ sandwich immunoassays by forming primary antibody (Ab_1_)/antigen (Ag, analyte)/labeled secondary antibody (labeled-Ab_2_) complexes (Ab_1_/Ag/labeled-Ab_2_), leading to a cumbersome operating process. In addition, the detection sensitivity of the first two methods is low. Although the latter has high detection sensitivity, it suffers from high detection costs because of the use of streptavidin-coated magnetic microbeads and ECL ruthenium complex-labeled antibodies. The above detection methods are also difficult to adapt to on-site detection. Electrochemical sensors have the advantages of rapid detection, high sensitivity, simple instrument, easy integration, and portability [10,11,12,13,14,15,16]. Therefore, electrochemical immunoassays offer advantages for sensitive, convenient, and even in-situ or point-of-care detection of PSA in serum.

A suitable supporting electrode is the basis for constructing electrochemical immunosensors. Since it is difficult to maintain excellent detection performance after regeneration in most immunosensors, the construction of disposable electrochemical immunosensors with inexpensive and one-use only electrodes is highly desirable [17,18]. This is attributed to the disadvantages of electrochemical immunosensors based on reusable electrochemical electrodes, such as high cost, complex fabrication, and difficulty in batch fabrication. For example, the most common renewable electrodes are noble metal electrodes (e.g., Au, Pt electrodes) and carbon electrodes (e.g., glassy carbon electrode (GCE), carbon paste electrodes, carbon fiber electrodes, etc.). However, these electrodes are expensive and often need to be polished with particulate slurries (such as 1 μm, 0.3 μm, and 0.05 μm Al_2_O_3_ slurry) before use, which is complicated to operate [19,20]. Disposable screen-printed electrodes (SPCE) are inexpensive and could be mass-manufactured. Several SPCE-based immunosensors were developed for PSA detection [21,22,23]. However, the fabrication of the recognitive interface in these sensors is usually complicated, and it is easy to contaminate the electrode surface. Recently, patterned indium tin oxide (ITO) electrodes attracted much attention as inexpensive and disposable electrodes [24,25,26]. ITO is prepared by doping high-valent Sn^4+^ into In_2_O_3_. A large number of free electrons resulting from the doping structure endow ITO with excellent electrical conductivity as an n-type semiconductor. Until now, many techniques (e.g., magnetron sputtering, chemical vapor deposition, sol–gel, electron beam evaporation, etc.) were used to prepare ITO films on different substrates (such as rigid glass, or flexible polyethylene terephthalate (PET), polyimide (PI), and so on) with firm bonding with the substrate, and scratch resistance. Therefore, ITO electrodes have the advantages of rigid or flexible structure, easy patterning, mass production, low cost, and excellent electrochemical performance, demonstrating great potential for constructing disposable electrochemical immunosensors.

The detection modes of electrochemical immunosensors include two categories [27,28]. One is to the directly or indirectly electrochemical signals generated by labels in the formed Ab_1_/Ag/labeled-Ab_2_ sandwich complex. The other type is to achieve label-free detection by redox probes in solution or immobilized on the electrode surface [29,30,31]. Briefly, the binding of antibodies towards the detected antigen on the immuno-recognitive interface changes the interface resistance of the electrode, which, in turn, leads to a change in the electrical signal of the probe. Amongst these, the detection based on solution-state probes has the advantages of convenient operation and simple electrode construction. The improvement in the detection sensitivity of this solution–probe-based immunosensor by introducing nanomaterials with signal amplification is crucial. Very recently, the remarkable signal amplification effect by vertically ordered mesoporous silica nanochannel film (VMSF) attracted much attention [32,33,34]. VMSF is a nanometer ultrathin film (50~200 nm in thickness) composed of silica nanochannels parallel to each other with high density (up to 3~12 × 10^12^ cm^−2^) and uniform pore size (usually 2–3 nm in diameter) [35,36,37]. On the one hand, the open and high-density nanochannel array ensures efficient diffusion of small molecules. On the other hand, the ultra-small nanochannels have an ultra-high specific surface area, showing excellent charge-based permselectivity [38,39,40]. The variability of VMSF structure endows it with flexible enrichment towards small molecules with different charges [40,41]. For example, the ionization of silanol groups (Si-OH, p*K*_a_~2) on commonly prepared VMSF using tetraethoxysiloxane (TEOS) as the precursor provides negative charge that can repel anions, but shows efficient enrichment towards cations [42,43,44]. When VMSF with rich amino groups (NH_2_-VMSF) is prepared using 3-aminopropyltriethoxysilane-APTES as a precursor, it has a large number of positively charged sites, leading to a significant attraction on negatively charged probes [45]. Therefore, VMSF-modified electrodes can significantly enrich small molecule probes in solution, improving the detection sensitivity of the electrochemical immunosensors. In addition, the size exclusion effect of ultra-small nanochannels can avoid the contamination of the electrode surface by the complex matrix (e.g., proteins) in biological samples [46]. Therefore, VMSF-modified disposable electrodes have great potential in the convenient and sensitive detection of PSA in serum.

Herein, a label-free electrochemical immunosensing platform was fabricated for sensitive detection of PSA in human serum based on modification of miniaturized, integrated, and disposable ITO electrodes with amino-rich nanochannel arrays (NH_2_-VMSF). When surfactant micelle (SM) and amino-containing siloxanes were used as template and precursor, respectively, NH_2_-VMSF was rapidly grown (<10 s) by electrochemical-assisted self-assembly (EASA) method. To achieve covalent immobilization of the recognitive antibody (Ab), the amino groups on the outer surface of NH_2_-VMSF reacted with bifunctional glutaraldehyde to generate aldehyde-based surfaces. The blocking of the nanochannels by SM ensures that the aldehydeylation occurs only at the entrance of the nanochannels and not within the nanochannels. The open nanochannel array after SM was removed exhibited remarkable enrichment towards anionic electrochemical redox probes (Fe(CN)_6_^3−/4−^) in the solution. Since the immunocomplex formed by the binding of Ab and PSA on the immuno-recognitive interface hindered the diffusion of Fe(CN)_6_^3−/4−^ to the underlying electrode, the immunosensor can realize sensitive detection of PSA. In comparison with the immunosensors with complicated fabrication process, our immunosensor has the advantages of simple fabrication and high sensitivity.

## 2. Materials and Methods

### 2.1. Chemicals and Materials

All reagents used in the experiment were of analytical grade without further treatment. Prostate-specific antigen (PSA), mouse anti-human PSA monoclonal antibody (Ab), carcinoembryonic antigen (CEA), and carcinoma antigen 125 (CA125) were purchased from Beijing KEY-BIO Biotech Co., Ltd. (Beijing, China). Bone gamma-carboxyglutamate protein (BGP) was purchased from Nanjing Okay Biotechnology Co., Ltd. (Jiangsu, China). S100 calcium-binding protein β (S 100) was obtained from Proteintech (Wuhan, China). Potassium ferricyanide (K_3_[Fe(CN)_6_]), potassium ferricyanide (K_4_[Fe(CN)_6_]), tetraethyl orthosilicate (TEOS), cetyltrimethylammonium bromide (CTAB), potassium hydrogen phthalate (KHP), glutaraldehyde (GA), and fetal bovine serum (BSA) were all purchased from Aladdin Biochemical Technology Co., Ltd. (Shanghai, China). Sodium nitrate (NaNO_3_) was obtained from Prospect Chemical Reagent Co., Ltd. (Wuxi, China). Additionally, 3-aminopropyltriethoxysilane (APTES) was obtained from Macklin biochemical Technology Co., Ltd. (Shanghai, China). Anhydrous ethanol and sodium hydroxide (NaOH) were purchased from Gaojing Fine Chemical Co., Ltd. (Hangzhou, China). Phosphate buffer (PBS, 0.01 M, pH 7) was prepared by Na_2_HPO_4_ and NaH_2_PO_4_. Deionized water (18.2 MΩ cm) was prepared by Mill-Q system (Millipore Company, Shanghai, China). ITO-coated glasses (<17 Ω/square, thickness: 100 ± 20 nm) obtained from Zhuhai Kaivo Optoelectronic Technology (Zhuhai, China) were first cleaned by NaOH aqueous solution (1 M), and subsequently sonicated in acetone, ethanol, and ultrapure water prior to use.

### 2.2. Measurements and Instrumentations

The morphology of NH_2_-VMSF was investigated by transmission electron microscope (TEM, JEM-2100, JEOL, Tokyo, Japan) with an acceleration voltage of 200 kV. Before measurement, the NH_2_-VMSF on the ITO electrode was scraped off slowly with a blade, dispersed evenly with ethanol, and dripped on the copper net. The morphology and thickness of NH_2_-VMSF were characterized by scanning electron microscope (SEM, SU8010, Hitachi, Tokyo, Japan) with an acceleration voltage of 5 kV. Before investigation, the electrode surface was scratched with a glass knife and divided into small pieces, the cross-section was placed upward and stuck on the sample table with conductive adhesive. Then, the sample was observed after spraying with gold. X-ray photoelectron spectroscopy (XPS) analysis was carried out on a PHI5300 electron spectrometer using 250 W, 14 kV, Mg Kα radiation (PE Ltd., Boston, MA, United States). Electrochemical impedance spectroscopy (EIS), cyclic voltammetry (CV), and differential pulse voltammetry (DPV) measurements were performed on an Autolab (PGSTAT302N) electrochemical workstation (Metrohm, Switzerland). A traditional three-electrode system was employed for electrochemicals. Briefly, bare or modified ITO was used as the working electrode, Ag/AgCl was used as the reference electrode, and a platinum wire electrode was used as the counter electrode. The scan rate used in cyclic voltammetry (CV) measurement was 50 mV/s, the parameters for DPV measurements included step potential (0.005 V), pulse amplitude (0.05 V), interval time (0.2 s), and pulse time (0.05 s).

### 2.3. Preparation of NH_2_-VMSF/ITO Electrode

As reported previously, NH_2_-VMSF was grown on the bare ITO electrode by using the electrochemically assisted self-assembly (EASA) method [47]. Briefly, APTES (0.318 mL) and 1.585 g CTAB (4.34 mM) were added to a mixture of 20 mL NaNO_3_ (0.1 M, pH 2.6) and 20 mL ethanol. When TEOS (2.732 mL) was added to the solution, it was necessary to adjust the pH to 3 with concentrated HCl. The solution was aged for 2.5 h under stirring before use. Then, the bare ITO electrode was immersed into the above solution and grown for 10 s with constant current density (−0.70 mA/cm^2^). After being quickly washed with ultrapure water, the obtained electrode was dried with nitrogen, aged at 120 °C overnight, and termed as SM@NH_2_-VMSF/ITO. SM template extraction was achieved by immersing the film electrode in an ethanol solution containing 0.1 M HCl under moderate stirring for 5 min, and the resulting electrode was termed as NH_2_-VMSF/ITO.

### 2.4. Fabrication of Label-Free Immunosensor

Owing to the presence of amino groups on NH_2_-VMSF, glutaraldehyde (GA) was chosen as the bifunctional linker for covalent immobilization of the recognitive bioligands. Firstly, the prepared SM@NH_2_-VMSF/ITO electrode was soaked in 5 % GA solution for 30 min at 37 °C in dark. After unlinked GA was washed off, the micelle was removed by stirring for 5 min in 0.1M HCl/ethanol (*V*: *V* = 1: 1) solution to obtain GA/NH_2_-VMSF/ITO. Then, to fabricate the immune recognition interface, the PSA antibody (50 μL, 10 μg/mL) was drop-coated on the surface of GA-NH_2_-VMSF/ITO. After incubation at 37 °C for 90 min, the unbound antibody was rinsed with PBS (0.01 M, pH 7). Finally, the obtained electrode was then incubated with BSA solution (1 %, wt%) for 60 min to block the non-specific sites, followed by rinsing with PBS. The prepared immunosensor was denoted as Ab/GA/NH_2_-VMSF/ITO and stored in a refrigerator at 4°C.

### 2.5. Electrochemical Determination of PSA

The Ab/NH_2_-VMSF/ITO immunosensor was incubated with different concentrations of PSA (antigen) at 37 °C for 45 min before electrochemical testing. KCl (0.1 M) containing Fe(CN)_6_^3−/4−^ (2.5 mM) was applied as the electrolyte solution. The electrochemical signal of the Fe (CN)_6_^3−/4−^ in the electrolyte was measured before and after PSA binding. Healthy human serum was diluted 50-fold with PBS (0.01 M, pH 7) for real sample analysis. To simulate the different PSA concentrations of prostate cancer patients, artificial PSA was added to the serum and then detected with the developed immunosensor.

## 3. Results and Discussion

### 3.1. Fabrication of Immunosensor on Amino-Rich Nanochannel Array-Modified Electrode

In recent years, researchers developed electrochemical-assisted growth method (EASA) or Stöber-solution growth method to induce the synergistic occurrence of surfactant molecular self-assembly and organosilane hydrolysis/polycondensation [48,49], which establish the growth of vertically ordered mesoporous silica nanochannel films (VMSF) on solid surfaces. VMSF has excellent properties, including ultra-thin and adjustable thickness, highly uniform pore size and nanochannel distribution, extremely high porosity, excellent mechanical/chemical/thermal stability and biocompatibility, and easy surface functionalization [50,51]. Moreover, the cost is low, and it can be prepared in batches in a large area, so it is an ideal electrode modification material. VMSF can be stably modified on indium tin oxide (ITO) electrodes [52,53]. This is attributed to the covalent bonding between VMSF and the ITO surface through the formation of -Si-O-In- or -Si-O-Sn- bonds. In this work, VMSFs containing amino groups (NH_2_-VMSF) were grown on the surface of patterned ITO electrode by the EASA method, which can grow VMSFs in a very short time (within 10 s). Although antibodies, as protein macromolecules, cannot enter the nanochannels of VMSFs, they can be easily immobilized on the outer surface of VMSFs, which is the entrance of the nanochannels. By introducing reactive groups (such as -NH_2_ groups) into VMSFs, the conversion of chemical groups can be flexibly achieved through covalent reactions.

**Figure 1:**
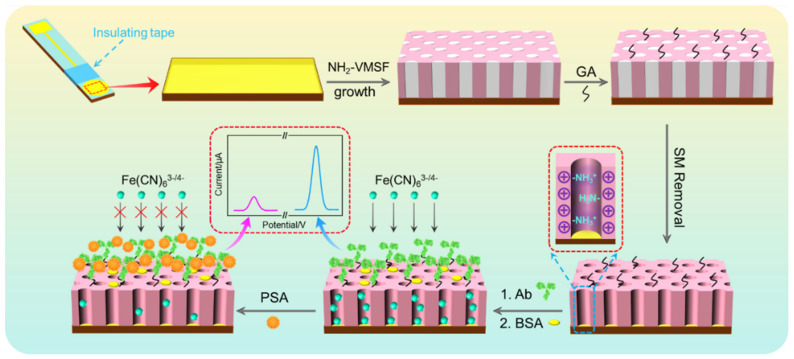
Schematic illustration for the fabrication of label-free immunosensor and the determination of PSA using immunocomplex-gated electrochemical signal.

As illustrated in Figure 1, the patterned ITO electrode consists of a square area as the working electrode and a thin linear section as the wire. Insulating tape is applied to the intersection of the thin wire and the working electrode area to ensure that different electrodes have a consistent electrode area. When NH_2_-VMSF is grown on ITO, SM remains within the nanochannels (SM@NH_2_-VMSF/ITO), thereby blocking the nanochannel. Then, aldehyde group derivatization is performed on the outer surface of NH_2_-VMSF to obtain a surface with aldehyde groups through reactions between amino groups and glutaraldehyde (GA). After removal of SM, the covalent immobilization of the recognitive antibody (Ab) is subsequently achieved through the reaction between aldehyde groups and amino groups in Ab. The immunosensor Ab/GA/NH_2_-VMSF/ITO is obtained after blocking non-specific sites on the electrode surface with bovine serum albumin (BSA). Since Ab can bind of the targeted PSA, the formed immunocomplex prevents the entry of Fe(CN)_6_^3^^−/4−^ probe in solution, resulting in a reduction in the electrochemical signal. Based on this mechanism, electrochemical detection of PSA can be achieved.

### 3.2. Characterization of Morphology and Structure of NH_2_-VMSF

**Figure 2:**
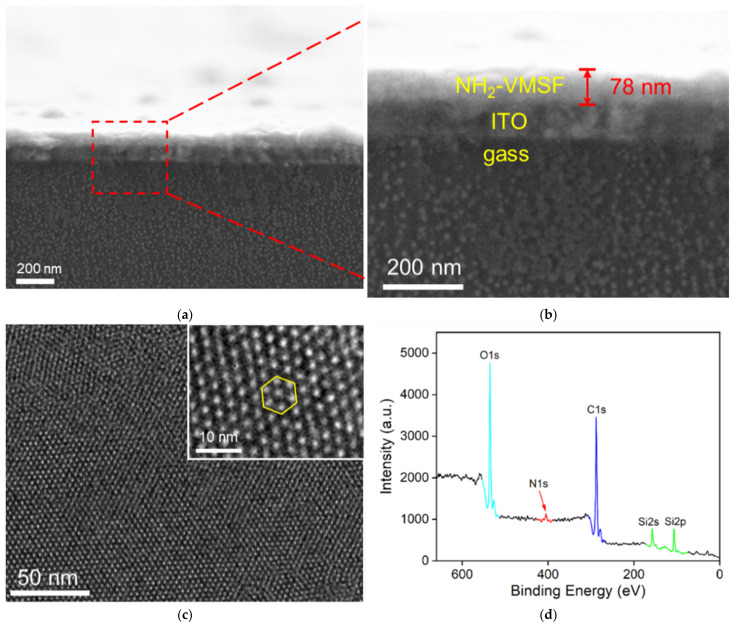
(**a**,**b**) The cross-sectional SEM image of the NH_2_-VMSF/ITO at different magnificence. (**c**) Top-view TEM image of NH_2_-VMSF at different magnifications. Inset is the HRTEM image. (**d**) X-ray photoelectron spectrum (XPS) of SM@NH_2_-VMSF/ITO.

The morphology of NH_2_-VMSF was characterized by scanning electron microscopy (SEM) and transmission electron microscopy (TEM). In Figure 2a, the cross-sectional SEM image of the NH_2_-VMSF/ITO shows the layered structure, including the glass layer, the ITO layer, and the NH_2_-VMSF layer. The thickness of NH_2_-VMSF is 78 nm (Figure 2b). Figure 2c is the top-view TEM image of NH_2_-VMSF after scraping off the electrode. As seen, NH_2_-VMSF has a continuous porous structure. There are no cracks in the larger area. The high-resolution TEM (HRTEM) image reveals a hexagonal packing structure (inset of Figure 2c). The pore diameter is between 2–3 nm. The pore density is ~7.5 × 10^12^/cm^2^, corresponding to a porosity of ~44 %.

NH_2_-VMSF was prepared using TEOS and APTES as mixed siloxanes. The functional groups on the outer surface of NH_2_-VMSF were applied for the fabrication of immuno-recognitive interface. To investigate the elemental composition of the outer surface of NH_2_-VMSF, X-ray photoelectron spectroscopy (XPS) of SM@NH_2_-VMSF/ITO was investigated. As shown in Figure 2d, in addition to C element (from SM), O and Si elements (from SiO_2_ structure) and the signal peaks of N also appear, proving that APTES introduces NH_2_ groups to NH_2_-VMSF. The active NH_2_ groups endow NH_2_-VMSF/ITO with great potential for further modification and functionalization.

### 3.3. Charge-Based Selective Permeability or Redox Probe in NH_2_-VMSF

**Figure 3:**
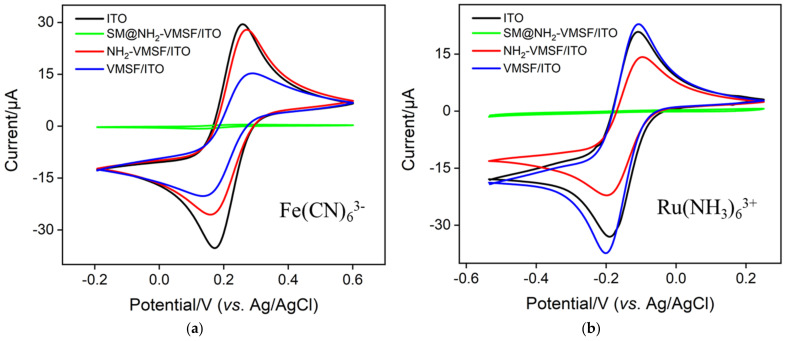
CV curves obtained from different electrodes in 50 mM KHP (pH 4) containing 0.5 mM Fe(CN)_6_^3–^ (**a**) and Ru(NH_3_)_6_^3+^ (**b**).

In the growth process of NH_2_-VMSF on ITO electrode, SM closed and open nanochannel array-modified electrodes are obtained successively. The integrity of NH_2_-VMSF and the penetration of probes were investigated by examining the electrochemical behavior of anionic (Fe(CN)_6_^3−/4−^, Figure 3a) and cationic (Ru(NH_3_)_6_^3+^, Figure 3b) standard redox probes on different electrodes. As controls, the supporting ITO electrode and VMSF-modified electrodes without amino groups (VMSF/ITO) were also examined. As shown, the redox peaks of the two probes are obvious on the ITO electrode. Despite the excellent electrochemical properties of ITO, its surface is difficult to derivatize directly. Furthermore, in practical applications, co-existing components in complex matrices tend to adhere to the ITO surface through non-specific adsorption, leading to fouling of the electrode and degraded electrochemical performance. In the case of the SM@NH_2_-VMSF/ITO electrode, no electrochemical signals are observed for either redox probes. This is due to the fact that the hydrophobic SM blocks the nanochannels, so that hydrophilic probes in solution cannot diffuse to the underlying electrode. This phenomenon proves that the grown VMSF is intact without cracks. When SM was removed to obtain an open nanochannel array, significant oxidation and reduction peaks of the two probes appear on the NH_2_-VMSF/ITO electrode. As shown, VMSF/ITO exhibits higher peak current in Ru(NH_3_)_6_^3+^ solution in comparison with ITO. In the employed pH of the electrolyte solution (pH 4), the ionization of abundant silanol groups (p*K*_a_~2) in VMSF make a negatively charged surface, leading to electrostatic adsorption effect on cationic probe Ru(NH_3_)_6_^3+^ and a higher CV signal. Compared with ITO, the electrochemical signal of NH_2_-VMSF/ITO recovers to some extent because of the reduced active surface after the growth of non-conductive NH_2_-VMSF. It is worth noting that NH_2_-VMSF/ITO shows different peak currents for both probes in comparison with that of VMSF/ITO. As is well-known, the ionization of abundant silanol groups (p*K*_a_~2) in VMSF make a negatively charged surface. The introduction of amino groups endows NH_2_-VMSF with positively charged sites. Due to the high surface area of the nanochannel arrays, the modified electrodes exhibit remarkable charge-based permselectivity for ion probes. Specifically, the electrostatic repulsion between the positively charged sites of NH_2_-VMSF and Ru(bpy)_3_^2+^ leads to a lower peak current on NH_2_-VMSF/ITO than that of VMSF/ITO. In the negatively charged probe solution, the situation is just the opposite. The signal on NH_2_-VMSF/ITO is higher than that of VMSF/ITO electrode due to the electrostatic attraction towards the negatively charge probe. Thus, the modification of NH_2_-VMSF facilitates the diffusion of negative Fe (CN)_6_^3−/4−^ to the underlying electrode.

### 3.4. Electrochemical Characterization of the Fabrication Process of Immunosensor

**Figure 4:**
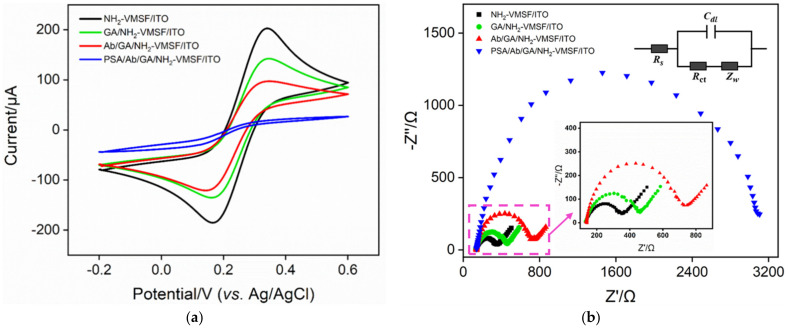
CV (**a**) and EIS (**b**) curves obtained on different electrodes. The electrolyte solution is 0.1 M KCl containing 2.5 mM Fe (CN)_6_^3−/4−^. Insets in (**b**) are equivalent circuits of detection (top inset) and the enlarged view of the EIS curves at the high frequency region (bottom inset).

Electrochemical methods including cyclic voltammetry (CV) and electrochemical impedance spectroscopy (EIS) were used to investigate the changes in the electrode surface during the fabrication of the immunosensor. Figure 4a shows the cyclic voltammetric curves of different electrodes. GA/NH_2_-VMSF/ITO was obtained by reacting SM@NH2-VMSF/ITO with GA and then removing SM. It can be seen that the peak current of redox probes on GA/NH_2_-VMSF is slightly reduced, which may be attributed to the cross-linking of partial amino groups by GA at the entrance of nanochannels. The peak current of the redox probe on the resulting immunosensor Ab/GA/NH_2_-VMSF/ITO further reduces when Ab is covalently immobilized on the aldehyde surface and the following blocking of non-specific binding sites by BSA. This is attributed to the fact that proteins act as non-conductive species to increase the interfacial resistance of the electrode surface. When the immuno-electrode is incubated with PSA, the probe signal significantly decreases due to the formation of antigen–antibody immunocomplexes that hinder the entry of the probe into the nanochannel, reducing the electrochemical signal of the probe. Similar conclusions are verified by EIS, as shown in Figure 4b, with the electron transfer resistance (*R*et) related to the semicircle diameter of each curve gradually increasing with GA modification, fabrication of immuno-recognitive interface, and PSA binding. The above results validate the efficient development of the immunosensor.

**Figure 5:**
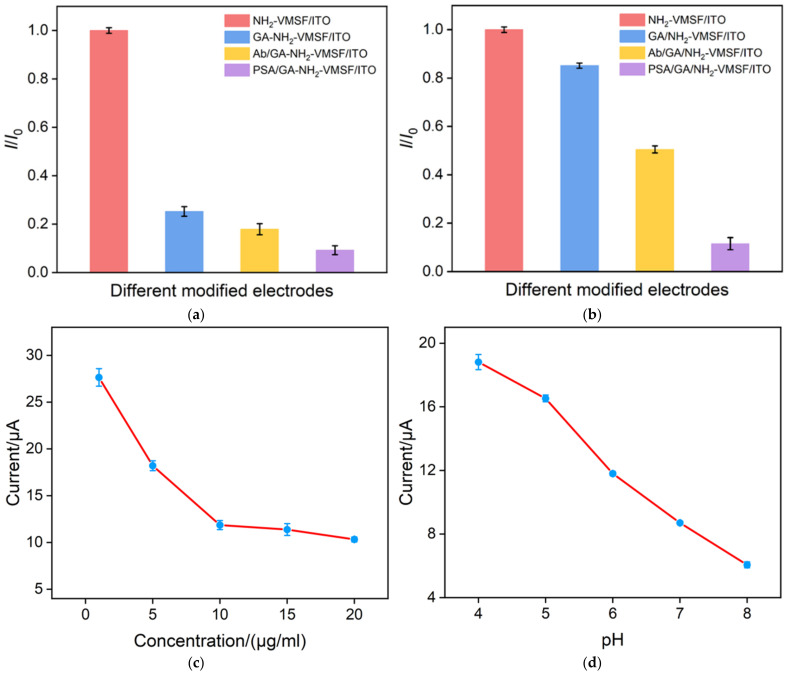
(**a**,**b**) Relative current values (*I*/*I*_0_) obtained at open (**a**) or SM-closed (**b**) nanochannel array-modified electrodes, where *I*_0_ is the peak current obtained on the electrode before GA modification, and *I* is the peak current obtained on the electrode after stepwise modification. (**c**,**d**) Peak current obtained in 0.1 M KCl solutions containing 2.5 mM Fe(CN)^3−/4−^ after GA/NH_2_-VMSF/ITO was incubated with different concentrations of antibody. (**b**) Peak current obtained on Ab/GA/NH_2_-VMSF/ITO in 0.1 M KCl solutions containing 2.5 mM Fe(CN)^3−/4−^ at different pH.

The aldehydeylation of the outer surface of NH_2_-VMSF is the basis for the construction of the immunosensing interface. The effect of aldehydeylation on the performance of the constructed immunosensors using open or SM-closed nanochannel arrays was investigated. Amongst these, the former was denoted as GA-NH_2_-VMSF after derivatization with GA. The electrochemical signal of Fe(CN)_6_^3−/4−^ on different electrode was determined by DPV. Figure 5a shows the relative current value (*I*/*I*_0_) obtained on different electrodes, where *I*_0_ is the peak current obtained on the electrode before GA modification, and *I* is the peak current obtained on the electrode after stepwise modification. As seen, the signal of the redox probe on the GA-NH_2_-VMSF/ITO electrode significantly reduces when the open nanochannel is employed for GA modification. This is attributed to the cross-linking of GA to the amino groups inside the nanochannel, which greatly reduces the diffusion of the probe to the underlying electrode. The immunosensor constructed by this strategy has a very low electrochemical signal. This results in the detection of PSA only in a narrow concentration range. In contrast, when the SM-blocked nanochannel array is aldehydeylated, the reaction is directed to the outer surface of NH_2_- because the nanochannels are filled with hydrophobic SM (Figure 5b). After SM removal, the open nanochannels effectively ensure the diffusion of probes. In addition, the immunosensor after binding with Ab still has a high electrochemical signal and the binding of PSA significantly reduces the electrochemical signal, which leads to sensitive electrochemical detection and a wide detection range. Therefore, in this paper, SM-blocked nanochannel arrays are used for the introduction of aldehyde groups to construct immunosensors.

The influence of the amount of antibody or pH value was investigated. Figure 5c reveals the peak current of GA/NH_2_-VMSF/ITO after incubation with different concentrations of Ab. When Ab concentration increases, the peak current of redox probe decreases and then reaches an almost stable value. When the antibody is fixed on the outer surface of VMSF, it closes the entrance of some nanochannels because of the large size, leading to reduced diffusion of redox probes into the nanochannels. The appearance of stable signals indicates that the amount of immobilized antibody is close to saturation. Thus, the concentration of Ab was chosen as 10 μg/mL. Figure 5d shows the effect of pH on the peak current of the redox probe on the immunosensor. The peak current increases with the increase in pH value. This is due to the charge change of amino groups under different pH conditions. With the increase in pH, the deprotonation of amino group leads to the gradual decrease in positive sites, weakening the adsorption towards negatively charged probes. Considering the stability of Ab and the neutral environment of serum samples, pH 6 was selected for further experiments.

### 3.5. Label-Free Electrochemical Determination of PSA

**Figure 6:**
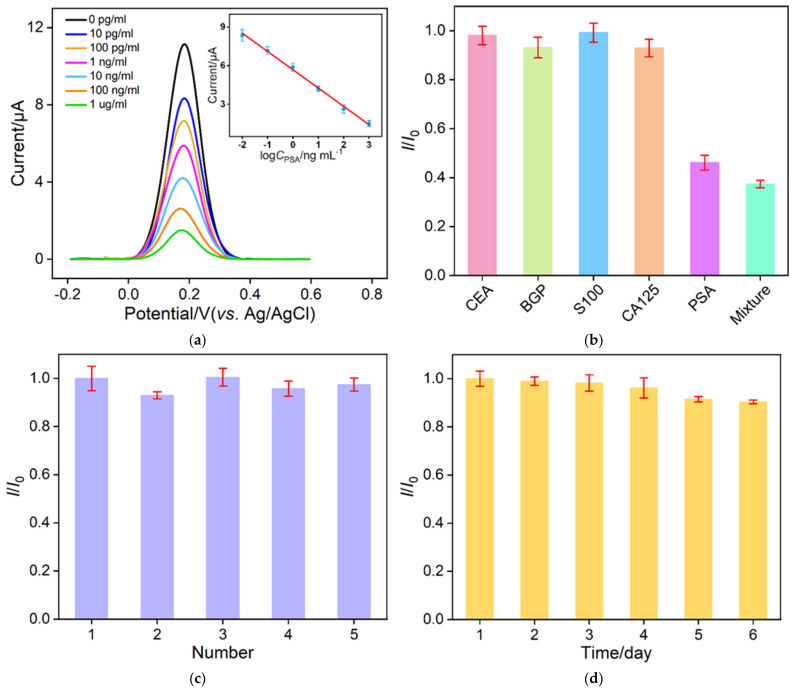
(**a**) DPV response of the immunosensor after incubation with different concentrations of PSA. The inset is the linear relationship between the peak current and the logarithmic value of PSA concentration. (**b**) Relative ratio of DPV peak current before (*I_0_*) and after (*I*) incubation with CEA (10 ng/ml), BGP (1 ng/mL), S100 (10 ng/ml), CA125 (1 μU/mL), PSA (10 ng/mL), or their mixture. (**c**) Relative ratio of DPV peak current obtained on five parallel electrodes. I0 and I are peak current obtained on the 1st or other electrodes. (**d**) Relative ratio of DPV peak current obtained on immunosensor stored at 4 °C for different times.

The performance of the constructed sensor in the electrochemical detection of PSA was investigated. The detection principle was the reduction in the electrochemical probe signal in solution after the specific binding of PSA and Ab. The immunosensors were incubated with different concentrations of PSA, and then the DPV curves of the electrodes were determined. As shown in Figure 6a, the DPV peak current of the immunosensor gradually decreases with increasing PSA concentration. The linear regression curve in inset in Figure 6a shows a good linear relationship between the peak current (*I*) and the logarithmic value of PSA concentration (log*C*_PSA_). The detection range is from 10 pg/mL to 1 ug/mL (*I* = −1.42log*C*_PSA_ + 5.68, *R*^2^ = 0.995). The limit of detection (LOD) calculated based on a three-fold signal-to-noise ratio (S/N=3) is 1.3 pg/mL. The LOD is lower than that obtained based on electrochemical detection using PtCu hollow nanoframes [54], metal-ions-functionalized gold nanoparticles-carbon nanospheres CNSs@AuNPs [55], nafion/graphene oxide/aldehyde methyl pyridine (Nafion/rGO/CHO-MP) [56], or metal organic frame-235/methylene blue (MOF-235/MB) [57]-modified electrode, but higher than that obtained based on palladium-nanoparticles-loaded electroactive amino-zeolitic imidazolate framework-67 (Pd/NH_2_-ZIF-67) [58], electrochemiluminescence (ECL) detection based on cadmium sulfide/chitosan/g-C_3_N_4_ (CdS/Chito/g-C_3_N_4_) [59], or electrodeposited gold@poly-luminol nanocomposite (Au@PL-NC) [60].

Excellent selectivity is crucial for the practical application of immunosensors. The selectivity of the constructed immunosensor was examined by detecting PSA or other interfering tumor biomarkers, including carcinoembryonic antigen (CEA), bone gamma-carboxyglutamate protein (BGP), S100 calcium-binding protein β (S 100), and carcinoma antigen 125 (CA125). Immunosensors were incubated with a single protein or a mixture of all proteins. As shown in Figure 6b, a significant reduction in electrochemical signal is observed only when the immunosensor is co-incubated with PSA and PSA-containing mixed proteins. The other tested proteins do not cause significant changes in the electrochemical signaling response. This indicates that the constructed immunosensor has high selectivity for PSA. Using the same experimental conditions, we prepared five sensing electrodes in parallel and measured the response to 50 ng/mL PSA to evaluate the reproducibility of the immunosensor construction process. As shown in Figure 6c, the five electrodes show close responses with a relative standard deviation of 3.4 %. Therefore, the electrode preparation process has high reproducibility. The storage stability of the immunoelectrode was also investigated. After storage of the immunosensor at 4 °C for 6 days, the response to 50 ng/mL PSA maintains 91.5 % of the initial signal, indicating the high storage stability of the immunosensor.

### 3.6. Real Sample Analysis

**Figure 7:**
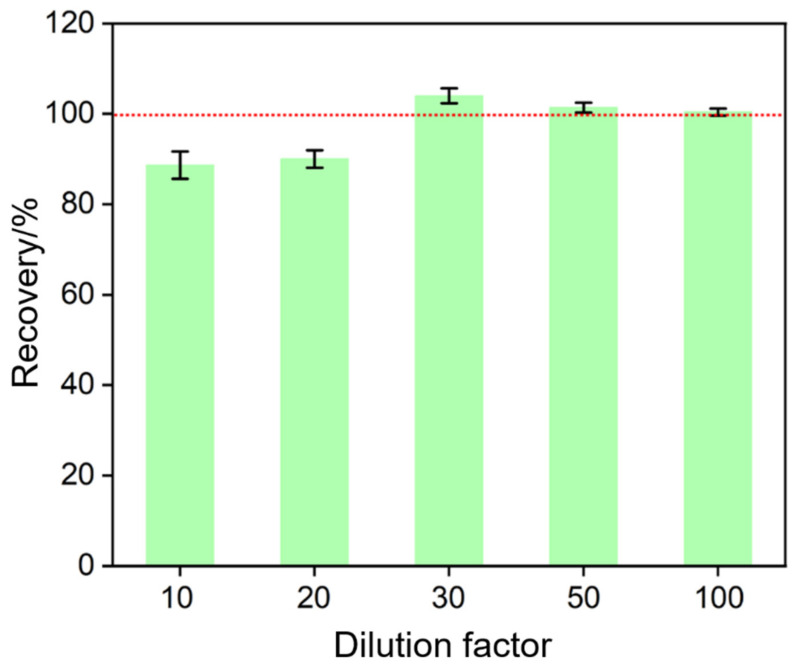
Recovery for the detection of PSA (10 ng/mL) in serum matrix when serum was diluted by different factors.

The practical application of the constructed immunosensor was evaluated by measuring the concentration of PSA in human serum. To investigate the effect of the serum matrix on the detection of PSA, PSA added in serum diluted by different factors was detected. As shown in Figure 7, the detection recovery is poor at a low dilution factor, which may be due to the influence of the viscous matrix on the diffusion of redox probes or the interference of coexisting substances. When the dilution factor is not less than 50 times, the serum matrix has no obvious effect on the detection recovery. The PSA concentration detected by the constructed immunosensor (1.25 ng/mL) is consistent with that (1.32 ng/mL) obtained by the commercial electrochemiluminescence analyzer. In addition, different concentrations of PSA were added to serum samples to mimic cancer patients with higher PSA concentrations. As shown in Table 1, the detected concentration of PSA recovery ranges from 95.7% to 103.2%, indicating good accuracy. Therefore, the immunosensor constructed in this paper has potential application in the clinical detection of PSA.

## 4. Conclusions

In summary, we constructed a disposable immunosensor for electrochemical detection of PSA in serum based on an array of amino-containing nanochannels modified on patterned ITO electrodes. Using amino-containing siloxane as a precursor, amino-rich VMSF (NH_2_-VMSF) was rapidly grown. Directed aldehyde group functionalization of the outer surface of NH_2_-VMSF followed by covalent immobilization of the antibody was performed when VMSF contained micelles to close the nanochannels. NH_2_-VMSF can facilitate the diffusion of negatively charged redox probes in solution to the underlying electrode. Sensitive electrochemical detection of PSA is achieved based on the hindrance of probe diffusion by immunocomplexes. The immunosensor constructed herein has the advantages of simple construction, sensitive detection, high reproducibility, and good stability. In addition, the ITO electrode used has low preparation cost, a highly adjustable electrode shape and area, and can be mass-produced. Therefore, the provided strategy is expected to be a universal electrochemical immunoassay platform for the convenient and mobile detection of tumor markers.

## Figures and Tables

**Table 1:** Determination of PSA in human serum.

Sample ^a^	Added (ng/mL)	Found (ng/mL)	RSD (%, n = 3)	Recovery (%)
Human serum ^a^	0.0100	0.0103	2.7	103.2
	0.100	0.0957	2.8	95.7
	10.0	10.1	3.7	101.4

^a^ Samples were diluted 50 times when PSA was added. The indicated concentration of PSA was obtained after dilution.

## Data Availability

The data presented in this study are available on request from the corresponding author.
